# The Flexible Armor of Chinese Sturgeon: Potential Contribution of Fish Skin on Fracture Toughness and Flexural Response

**DOI:** 10.3390/biomimetics8020232

**Published:** 2023-06-02

**Authors:** Yu Zheng, Xin Li, Ping Liu, Ying Chen, Ce Guo

**Affiliations:** 1 College of Mechanical and Electrical Engineering, Suqian University, Suqian 223800, China; yuzhengnuaa@163.com (Y.Z.);; 2 Institute of Bio-Inspired Structure and Surface Engineering, College of Mechanical and Electrical Engineering, Nanjing University of Aeronautics and Astronautics, Nanjing 210016, China

**Keywords:** fish skin, mechanical tests, fracture toughness, flexural stiffness, external tendon

## Abstract

Fish skin is a biological material with high flexibility and compliance and can provide good mechanical protection against sharp punctures. This unusual structural function makes fish skin a potential biomimetic design model for flexible, protective, and locomotory systems. In this work, tensile fracture tests, bending tests, and calculation analyses were conducted to study the toughening mechanism of sturgeon fish skin, the bending response of the whole Chinese sturgeon, and the effect of bony plates on the flexural stiffness of the fish body. Morphological observations showed some placoid scales with drag-reduction functions on the skin surface of the Chinese sturgeon. The mechanical tests revealed that the sturgeon fish skin displayed good fracture toughness. Moreover, flexural stiffness decreased gradually from the anterior region to the posterior region of the fish body, which means that the posterior region (near the tail) had higher flexibility. Under large bending deformation, the bony plates had a specific inhibition effect on the bending deformation of the fish body, especially in the posterior region of the fish body. Furthermore, the test results of the dermis-cut samples showed that the sturgeon fish skin had a significant impact on flexural stiffness, and the fish skin could act as an external tendon to promote effective swimming motion.

## 1. Introduction

Fish skin is covered by fish scales and connected with the underlying muscle tissue, unlike the biological structure, which has significant protective characteristics of fish scales. Therefore, its mechanical properties are often neglected. Previous studies have shown that fish skin is a biomaterial with multiple applications [1]. For example, gelatin in fish skin is the main component of biodegradable materials (such as gelatin film), which are more environmentally friendly than plastics [2,3,4]. Many fish skin cancer models established by researchers have demonstrated a high similarity with human skin diseases. With the rapid development of advanced molecular genetics, fish skin cancer models will continue to be improved and will provide important insights for future treatment methods [5,6,7]. Furthermore, fish skin is also regarded as a high-grade biological leather that has good application prospects [8,9].

Recent studies have shown that overlapping fish scales can resist predator attacks by producing a strain-stiffening effect, and this effect can be adjusted by the bending response of the fish skin, which reveals that the fish skin has good deformation and protective functions [10]. Szewciw et al. studied the structural and mechanical properties of striped bass (*Morone saxatilis*) fish skin and revealed (through tensile and penetration tests) that the stratum (s.) compactum is a critical tissue for the protective functions of fish skin [11]. The s. compactum in fish skin is mainly composed of the collagen matrix and fiber layers, and it may be a kind of strong tissue similar to the tendon. Kenley et al. conducted structural mechanical tests on the skin of three different fish living in the sea, namely coho salmon (*Oncorhynchus kitch*), Florida pompano (*Trachinotus carolinus*), and red snapper (*Lutjanus campechanus*) [12]. Their results showed that the helical fiber angles and tensile mechanical response of the s. compactum in fish skin varied in different body regions. The authors further revealed that s. compactum plays an essential role in fish movement and protective functions and significantly improves the biomechanical properties of fish skin. Furthermore, Zheng et al. studied the mechanical protective behaviors of the fish skin of three different fish species living in different water areas, namely Taihu white fish (*Erythroculter ilishaeformis*, from freshwater), grouper (*Cichlasoma managuense*, which live in both freshwater and seawater), and yellowfin seabream (*Ditrema temminckii Bleeker*, from the ocean) [13]. They found that for the three fish species, s. compactum plays a vital role in the protective behaviors of the fish skin. They also found that the helical fiber angles in s. compactum gradually decreased from the anterior region to the posterior region of the fish body. Their article further discusses the influence of the change of fiber angles on the protective mechanism of fish skin and supports that fish skin has significant mechanical protective functions.

The fish skin covers the body and is connected to the muscle tissue. During swimming, the fish skin is stretched and compressed, which means that the skin can directly transmit force and improve the swimming efficiency of the fish [14,15,16]. The flexural response of fish skin plays an essential role in the strain stiffening and progressive interlocking effects of fish scales [10]. Therefore, fish skin probably provides a potential tendon effect. Szewciw et al. conducted a bending test on the skin of striped bass (*Morone saxatilis*) and found that the flexural response was nonlinear [11]. Compared with the anterior and posterior regions of the fish body, the flexural stiffness of the middle region of the fish body is the highest. Their results showed that fish skin can store strain energy at high flexural stiffness and improve motion efficiency at low flexural stiffness. Moreover, fish skin can effectively reduce the tail beat frequency and propulsion wave speed during swimming by adjusting the stiffness of the body. Their incision experiment showed that the incisions they made significantly reduced the flexural stiffness of the fish body. Therefore, s. compactum plays a vital role in flexural stiffness, thus proving the potential tendon effect of fish skin.

Szewciw et al. only focused on striped bass (teleost fish) [11]. However, there may be differences in the flexural response and tendon effects of fish skin among different fish species. In this study, we examined the toughening mechanism of fish skin in Chinese sturgeon (cartilaginous fish) and the potential tendon effect of the skin and bony plates. Unlike most fish species, the body surface of Chinese sturgeon is only covered by a few rows of bony plates, and most regions of the body are covered only with fish skin. Compared with the scales of striped bass [17], the bony plates of Chinese sturgeon have a higher mineralization degree and strength [18,19]. We present a detailed analysis of the flexural response mechanism of Chinese sturgeon fish skin by tensile fracture and fish body bending tests and hope to explore its potential value in designing flexible and rigid protective structures.

## 2. Materials and Methods

### 2.1. Materials

**Figure 1:**
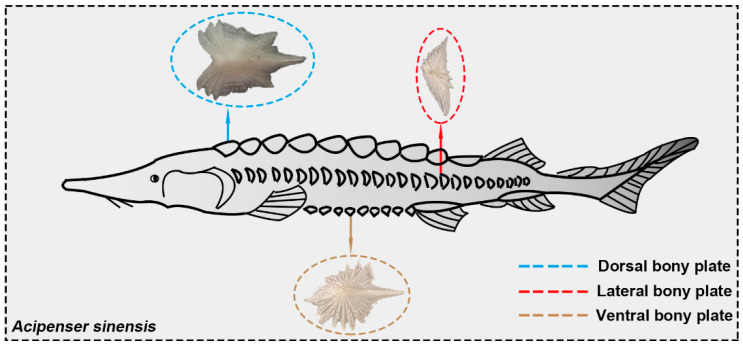
Morphological characteristics of Chinese sturgeon. There are three kinds of bony plates on the sturgeon body surface: dorsal bony plates, lateral bony plates, and ventral bony plates.

Seven fresh (recently deceased and on ice) Chinese sturgeons (captive-bred) were acquired from a local fish store (Nanjing, China). The weights and lengths of the fish were about 900 g and 400 mm, respectively. There are only five rows of bony plates on the body surface of the Chinese sturgeon: one row of dorsal bony plates, two rows of lateral bony plates, and two rows of ventral bony plates (Figure 1). Unlike common fish scales with high overlap rates, there was no overlap between the lateral bony plates and the ventral bony plates of the Chinese sturgeon.

Before the experiments, the Chinese sturgeons were washed with fresh water and soaked in fresh water for 20 min to bring them to room temperature (~25 °C). The skin samples of the two sturgeons were dissected with a scalpel and tweezers for microstructure observation. Additionally, the skin samples of two fish were dissected with a scalpel and tweezers and then soaked in freshwater for tensile fracture tests. The other three fish samples were prepared for bending tests. The samples prepared for scanning electron microscopy (SEM) observation were sealed in labeled plastic bags and preserved in a fridge at 3 °C. The tensile fracture and bending tests were started within 2 h after the fish samples were obtained. The tests performed enabled us to gain a preliminary understanding of the material and flexural properties of sturgeon fish skin. SEM observation was performed at the Nanjing Mechanical and Electrical Hydraulic Engineering Institute. Tensile fracture experiments were performed at the Nanjing University of Aeronautics and Astronautics.

### 2.2. Morphology Observation

Before the observation, the prepared skin samples were soaked in fresh water for 20 min (still in labeled plastic bags) to bring them to room temperature (∼25 °C). Then, the samples were removed from the plastic bags and dried with a vacuum freeze-drying technique. Finally, the square-shaped skin samples (*n*  =  10) with a length of 10 mm were processed using a sharp scalpel. The SEM (QUANTA 650, FEI: Portland, OR, USA) test was performed at 20 kV.

### 2.3. Tensile Fracture Experiments

**Figure 2:**
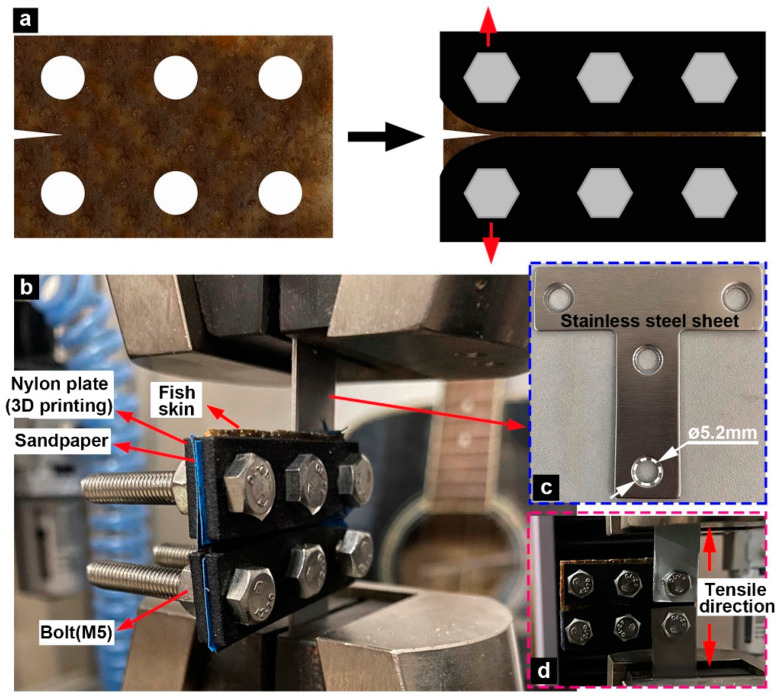
Tensile fracture tests. (**a**) Preparation and testing of the fracture sample. (**b**) The fixture settings for the sample comprise 3D-printed nylon plates, a bolt (M5), and (**c**) a stainless steel sheet. (**d**) Indication of the tensile direction.

Because fish skin is soft and thin, conventional fracture test methods (such as three-point bending) cannot achieve the experimental purpose. To solve this problem, a set of micro fixtures was prepared. The fixtures were composed of 3D-printed nylon plates, stainless steel sheets, and bolts (M5) (Figure 2b). Sandpaper (400 mesh) was added between the fish skin and the nylon plates to prevent significant slippage during the tests. Before the tests, three holes with a diameter of 5 mm were cut using surgical scissors and tweezers at the upper and lower ends of the fish skin, respectively. Additionally, a notch of 5 mm was cut using surgical scissors at one end of the fish skin (Figure 2a). Finally, fixation was achieved using bolts, nuts, and gaskets. One end of the stainless steel sheet was connected with the fixtures, and the other was fixed on the tensioning equipment. The number of test samples was *n* = 5, and the tests were performed on an electronic universal testing machine (CMT 4503, Wance Co., Ltd., Shenzhen, China) with a speed of 1 mm/min.

### 2.4. Bending Tests

**Figure 3:**
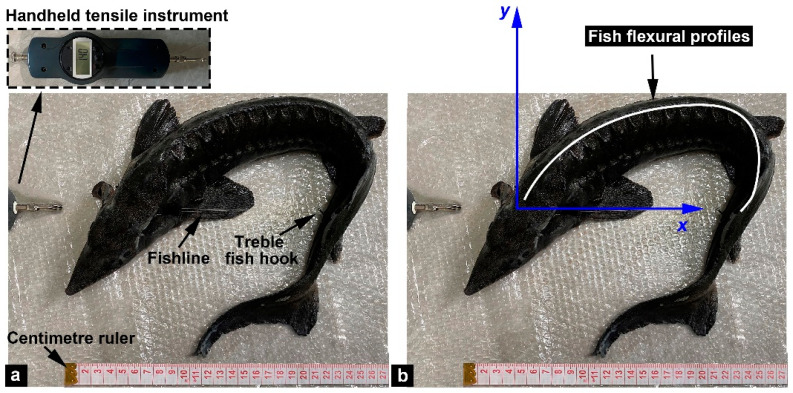
Bending tests. (**a**) Experimental setup for bending tests on whole sturgeon fish. (**b**) The (*x*, *y*) coordinate system is used to describe fish flexural profiles.

In the experiments, fish wire and handheld tensile instruments were used to measure the bending force of the fish body (Figure 3). First, we removed the opercular bones on the pharyngeal region on both sides of the fish to expose the gills and pharyngeal cavity. Then, a treble fish hook was inserted into the fishtail. Finally, the fish wire was carefully fed through the fish’s pharyngeal cavity from the left to the right side of the gills and connected to a tensile instrument (Weidu Co., Ltd., Wenzhou, China). The selected fish wire was lightweight and high strength, and the selected tensile instrument had enough accurate resolution (0.01 N). Experimental materials and equipment can effectively help us study the bending properties of the fish body. Moreover, a Panasonic digital camera (model no. DMC-GX85WK) was positioned over the fish body to take high-resolution images of the whole fish at each bending position for image analysis.

Before the bending tests, the fish were in a nearly straight body position, and then the fish wire was pulled until a high body curvature (such as the head contacting the tail) was achieved. Each test recorded 10 bending positions (with 10 associated images). The range of the 10 bending positions was from the nearly straight body position to the maximum bending position. It should be pointed out that the maximum bending amplitude of the fish body approached bending that occurs during sharp turns or escape responses, which is significantly greater than the bending amplitude of the fish during normal swimming [20]. To verify repeatability, we conducted two tests on the same fish. After completing the first two tests, the bony plates were removed using tweezers, and then for another two tests. Finally, a surgical knife was used to make 14 vertical incisions on the skin (on both sides) along the anteroposterior direction (incision length ~38 mm, and anteroposterior spacing ~24 mm), and the dermis-cut sample was tested twice. It should be noted that after each test, the sturgeon fish was immersed in fresh water for 15 min to avoid dehydration of the fish body, which could affect the accuracy of the bending tests. Compared to living fish, some tissue degradation may occur in the prepared fish samples. We also tested two other fish samples using the same conditions to test for possible degradation of properties during the bending tests. The results showed almost no degradation of properties during the tests. In addition, we established the coordinate system (*x*, *y*) to produce a series of fish flexural profiles (Figure 3b) for the captured pictures, where the *x*-axis aligned with the tensile direction and the origin corresponded to the intersection between the axis of the fish wire and head.

## 3. Results

### 3.1. Microstructural Characterization

**Figure 4:**
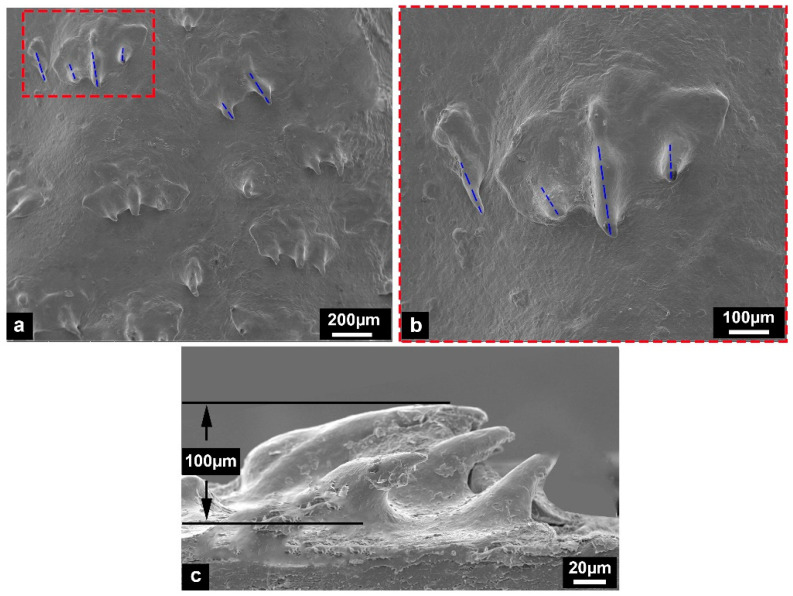
The morphological characteristics of the sturgeon fish skin surface. (**a**,**b**) Distribution of placoid scales on the fish skin surface, where the red frame and the blue lines shows the morphological details of the scales. (**c**) The lateral contour of placoid scales.

Although the bony plates did not cover most regions of the sturgeon skin surface, the SEM observation results showed some placoid scales on the skin surface (Figure 4). Researchers have found that the placoid scale is not a protective structure but a drag-reduction structure [21,22]. The ridge-like ribs on the surface of placoid scales can improve the fluid structure and flow state of the turbidity boundary layer and possess a good drag-reduction effect. Different from the placoid scales of the sharks [23], there was no overlap between the placoid scales of the Chinese sturgeon. Moreover, the number of scales was small, and their arrangements were irregular. Furthermore, ridge-like ribs with a height of −100 µm (Figure 4c) on the surface of the placoid scales were marked by blue dashed lines (Figure 4a,b). The number of ribs ranged from 1 to 3, and the smaller the number of ribs, the smaller the placoid scales. This was significantly different from the regular placoid scales on the skin surface of the sharks. The results showed that the placoid scales on the skin surface of Chinese sturgeon were probably degenerate structures.

### 3.2. Tensile Fracture Response

**Figure 5:**
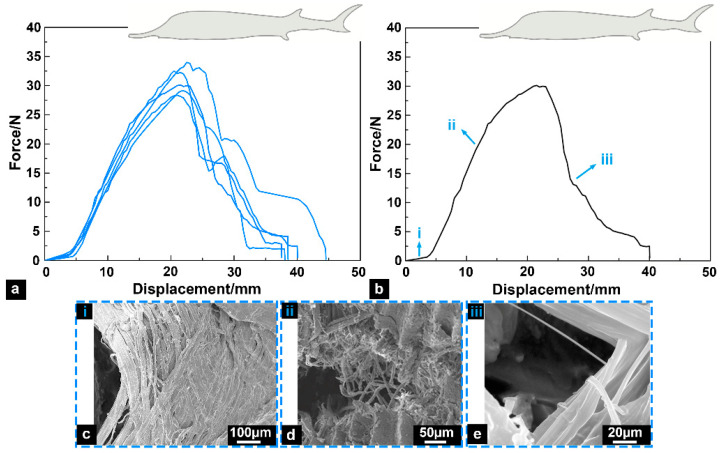
Tensile fracture tests. (**a**) Force-displacement curves of fish skin samples. (**b**) Different stages correspond to the curve. (**c**–**e**) SEM micrographs of fish skin at different stages.

The force-displacement curves for tensile fracture tests of fish skin samples are shown in Figure 5a. The experimental results demonstrated that the test curves presented a bell shape [24], indicating that the Chinese sturgeon skin had significant toughness characteristics. The average value of the maximum force that the fish skin samples could bear was 28.39 ± 3.15 N. The area under the force-displacement curves indicated the “fracture work” of the fish skin specimens. Then, the “fracture work” was divided by the surface area of the skin samples to obtain the energy required for the crack to expand on the unit area, which could be approximately regarded as the fracture toughness of the skin specimens. The average fracture toughness of the fish skin samples was 22.67 ± 1.23 kJ/m^2^, which was in the same order of magnitude as the cortical bone (~10 kJ/m^2^) [25] and some tough mammalian skin (~20 kJ/m^2^) [26], indicating that the Chinese sturgeon skin had superior fracture toughness.

The SEM observations found that the mechanical response of the skin samples in the tests could be summarized into three stages (Figure 5b–e):

(i) In the early stage of stretching, the skin samples had significant fiber delamination characteristics;
(ii) The tensile loads were significantly increased, and the skin samples mainly resisted the loads by fiber fracture;
(iii) At the later stage of stretching, a small amount of remaining fibrous tissues fractured, and the tensile loads decreased significantly.

The results revealed that the fiber layers in sturgeon fish skin displayed different toughening mechanisms to resist various loads. When the loads were small, the failure mode of the fiber layers was mainly fiber delamination, which could effectively delay the structural failure. However, when the loads increased significantly, the fibers started to break gradually, which markedly enhanced the fracture toughness of the fiber layers.

### 3.3. Flexural Profile Analysis

**Figure 6:**
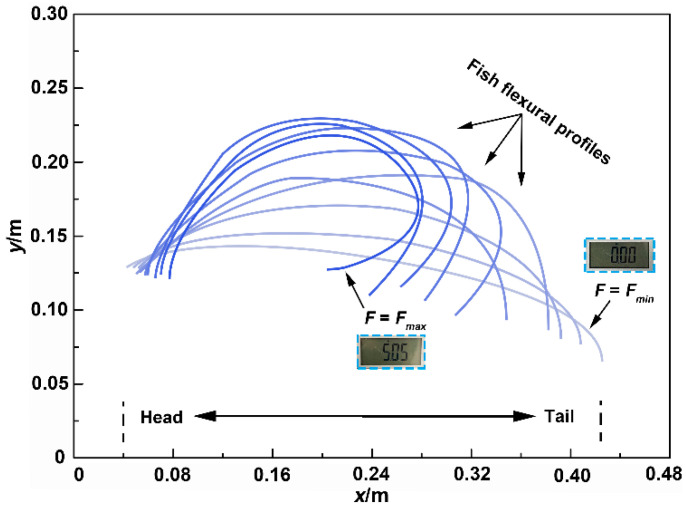
Fish flexural profiles in the (*x*, *y*) coordinate system. Profile curves for increasing force increments are shown from the right (zero force) to the left (maximum force).

**Figure 7:**
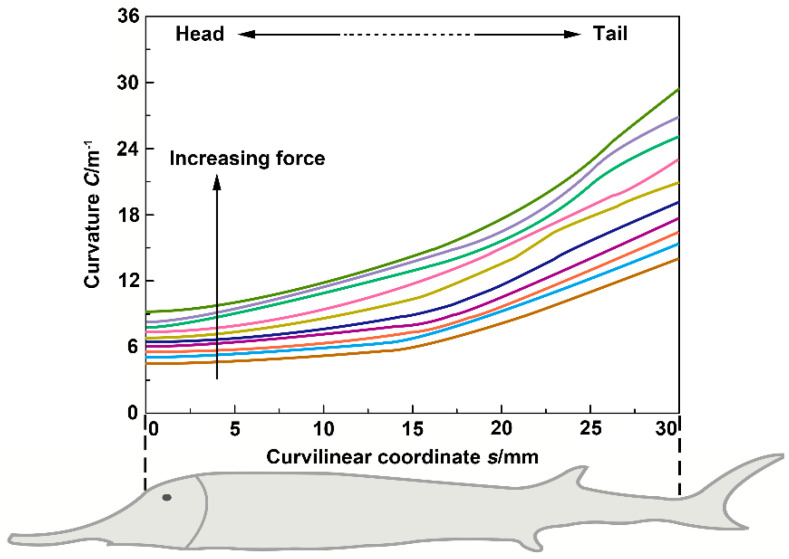
The curvature *C* of the fish body as a function of curvilinear coordinate *s*, where the different colored lines denote different force (F).

Figure 6 shows the flexural profile curves of the Chinese sturgeon in the bending tests. In the initial stage of the tests, the tensile force was *F* = 0. With the increased bending amplitude of the fish body, the distance (*x* value) between the head and tail of the fish decreased, while the *y* value and tensile force (*F*) increased. It can be seen that as the tensile force increased, the bending degree at each position of the fish body was different. Furthermore, each profile curve in Figure 6 was divided into six long segments, on average, and an arc of a circle was fitted onto a long segment of the profile curve. The curvature (*C*·m^−1^) was obtained by calculating the inverse of the circle’s radius, and then the curvature *C* was determined as a function of the curvilinear coordinate *s*. Figure 7 displays the curvature *C* of the fish body as a function of position along the fish (curvilinear coordinate *s*). The curvature *C* near the fish head was the lowest, while the curvature *C* near the fish tail was the highest. This demonstrated that the bending degree and flexibility near the fishtail were higher than those of the anterior and middle regions of the fish body.

### 3.4. Bending Moment versus Curvature

**Figure 8:**
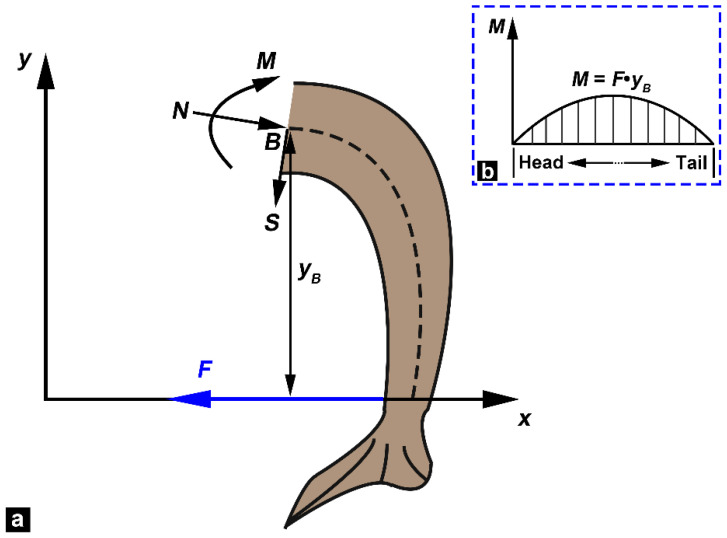
Calculation of the bending moment of the fish body. (**a**) Internal loads at point B: normal force *N*, shear force *S* and bending moment *M*, and the tensile force in the fish wire is *F*. (**b**) Changes in bending moment along the position of the fish body, where the bending moment is zero at the head and tail of the fish body.

The bending moment in the tests was obtained from the conventions used for bending beams (Figure 8). Internal loads should balance the applied tensile force F within the fish, including normal force *N*, shear force *S*, and bending moment *M* (Figure 8a). In this study, we assumed that the normal and shear forces induce negligible deformation compared with the bending generated by moment *M*. Therefore, only the moment *M* is considered, and its calculation formula is given by *M* = *F*·*y_B_*, where *y_B_* is the vertical coordinate of point *B*. The bending moment was zero at the head and tail of the fish body (Figure 8b). The middle region of the fish body is subjected to the highest bending moment because it is the furthest from the *x*-axis.

**Figure 9:**
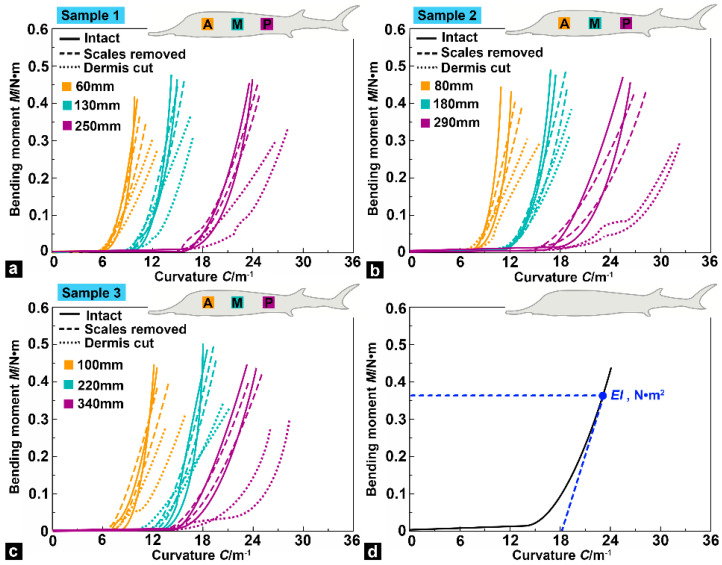
Bending moment versus curvature at three locations along the anteroposterior direction of the fish body. (**a**) *M*–*C* curves of sample 1 at 60 mm in the anterior region, 130 mm in the middle region, and 250 mm in the posterior region for sample 1. (**b**) at 80 mm, 180 mm, and 290 mm for sample 2. (**c**) at 100 mm, 220 mm, and 340 mm for sample 3. (**d**) Schematic descriptions of the *M*–*C* curve exhibiting tangent flexural stiffness *EI*.

Figure 9 shows the response curves of the bending moment (*M*/N·m) and curvature (*C*·m^−1^) at different positions of the fish body. The fish body was divided into three regions (curvilinear distances): anterior region (0–110 mm), middle region (110–230 mm), and posterior region (230–360 mm). Figure 9 shows the *M*–*C* curves at three locations of the fish body (curvilinear distances): at 60 mm in the anterior region, 130 mm in the middle region, and 250 mm in the posterior region for sample 1 (Figure 9a), and at 80 mm, 180 mm, and 290 mm for sample 2 (Figure 9b), and at 100 mm, 220 mm, and 340 mm for sample 3 (Figure 9c). The results showed that the required force to bend at a low curvature is negligible, which means the fish is highly flexible. However, at high curvature, significant stiffening was observed. Moreover, for the intact samples, samples (with scales removed), and dermis-cut samples, a rightward shift of the *M*–*C* curves along the anteroposterior direction of the fish body demonstrates that the posterior region (nearer to the fishtail) possesses a higher deformation ability compared with the other regions.

The average maximum tangent flexural stiffness (*EI* in N·m^2^, Table 1) was obtained by calculating the maximum slope of the *M*–*C* curves (Figure 9d) to analyze the differences of test samples under three different conditions. Compared with the intact samples, the average maximum tangent flexural stiffness of the samples (with scales removed) decreased by 6.38%, 2.78%, and 14.29% for sample 1; 4.44%, 6.06%, and 18.75% for sample 2; and 4.55%, 3.22%, and 17.65% for sample 3 in the anterior, middle, and posterior locations, respectively. These data showed that the influence of the bony plates on the flexural stiffness of the anterior and middle regions in the sturgeon body was minimal, but the influence of the posterior region was slightly increased. In addition, the average maximum tangent flexural stiffness of the dermis-cut samples showed a significant decrease compared with the samples (with scales removed): 36.36%, 45.71%, and 61.11% for sample 1, 39.53%, 48.39%, and 76.92% for sample 2, and 45.23%, 53.33%, and 71.43% for sample 3 in the anterior, middle, and posterior locations, respectively. The flexural stiffness at all three locations of the fish body decreased notably, and the declining trend was most prominent in the posterior region. The results reveal that s. compactum in sturgeon fish skin bears a significant portion of the bending moment. According to previous research [27], s. compactum in sturgeon fish skin endows the skin with superior mechanical properties. Therefore, the flexural stiffness of the fish body was significantly reduced after the s. compactum was cut. Furthermore, flexural stiffness gradually decreased along the anteroposterior direction of the fish body, which indicated that the deformation resistance in the body’s anterior region was higher than in other regions. This changing trend is different from that of the flexural stiffness of striped bass [11], which may be related to the shape of the fish’s body (such as the body cross-section diameter). The primary purpose of this test was to uncover the potential contributions of bony plates and s. compactum to the bending deformation of sturgeons and not to study the precise mechanical properties of the flexural stiffness of the fish body.

### 3.5. Flexural Response Characteristics

**Figure 10:**
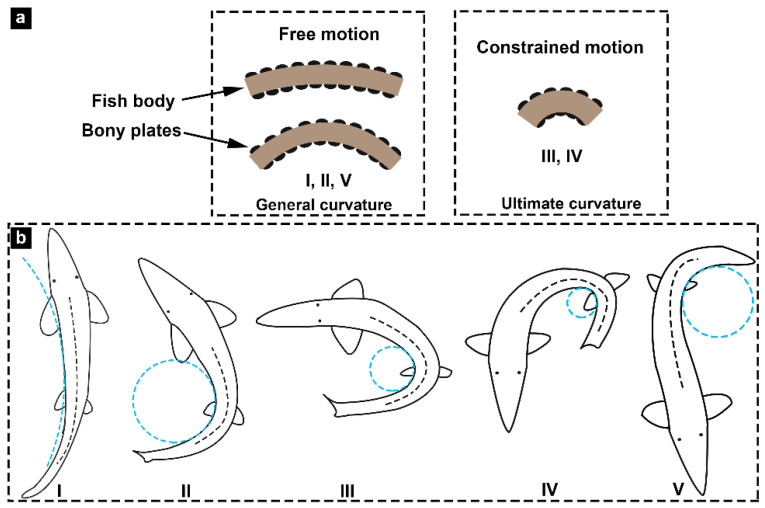
Effects of bony plates on bending deformation of Chinese sturgeon. (**a**) Bony plates versus bending motion. (**b**) Functional differentiation is adapted to the different radii of curvature (light blue dashed line) during swimming of the sturgeon fish, where the Roman numerals in caption show different degrees of deformation of fish bodies.

According to the analysis results of the bending tests and the flexural stiffness of the fish body, we drew and illustrated the effects of bony plates on the bending motion of Chinese sturgeon (Figure 10). In low and moderate bending deformations (I, II, and V in Figure 10), non-overlapping bony plates did not affect the bending motion of the fish body. In high bending deformations (III and IV in Figure 10), the bony plates on the inner, concave side of the fish body slightly affected the bending motion of the fish body. The curves of the fish flexural profile displayed that the bending amplitude near the tail of the sturgeon fish was significantly greater than that of the anterior and middle regions of the fish body, which revealed that the reason why the influence of the bony plates on the flexural stiffness of the posterior region of the fish body was more significant than that of the anterior and middle regions. In addition, the bony plates of the Chinese sturgeon were more mineralized compared with those of common fish scales, which meant that the bony plates possessed higher strength, hardness, and deformation resistance. This also explained why there was no overlap between the bony plates of Chinese sturgeon, that is, to avoid significantly increasing the difficulty of bending deformation of the fish body.

## 4. Discussion

This study used SEM observation, mechanical tests, and computational analysis to reveal the surface morphology, toughening mechanism, and potential tendon effects of Chinese sturgeon fish skin. Most regions of the sturgeon fish body were only covered by skin, and placoid scales covered the skin surface with different shapes and irregular arrangements. The placoid scale is a typical drag reduction structure and does not possess protective functions. Moreover, the morphology and distribution characteristics of the placoid scales on the sturgeon skin surface revealed that the scales were probably degenerate structures. The results of tensile fracture tests showed that sturgeon fish skin was an elastoplastic biological material. The fish skin started to produce elastic deformation at the initial stretching stage. With tensile loads increasing and exceeding the skin’s elastic limit, the fish skin generated partial plastic deformation, and the fiber layers in s. compactum appeared to have fiber delamination. As the loads continued to increase, the plastic strain of the fish skin increased sharply, and then a fiber fracture occurred. Because the intrachain bonds of fibrils are covalent and much stronger than the van der Waals or hydrogen in the interfiber bonds, the required loads for fiber delamination are much less than those required for fiber fracture [28]. The delamination, bridging, and fracture of fiber layers are common toughening mechanisms in biological materials, which can effectively disperse loads and delay structure failure [29].

The bending test results showed that flexural stiffness varied in different regions of the Chinese sturgeon body. The flexural stiffness of the posterior region (near the tail) was the smallest, which means that this region was more flexible. The flexural stiffness of the anterior region (near the head) was the largest, which means that this region was stiffer. This is likely to have a causal relationship with the thicker anterior regions (such as the larger body cross-section diameter) of the fish body. For example, previous studies have revealed that the middle region of the striped bass is thicker than the anterior and middle regions and demonstrates the largest flexural stiffness [30]. However, for the alligator gar, whose body is similar to a cylindrical shape, the thickness differences in the anterior, middle, and posterior regions are small, and the flexural stiffness along the anteroposterior direction of the fish body is relatively uniform [31]. In addition, research on striped bass revealed that the influence of fish scales on the bending deformation of fish is negligible. However, the results of this study showed that the bony plates of the sturgeons had little effect on the moderate bending deformation of the fish body but had a specific inhibition effect on the large bending deformation. Although the inhibition effect would hinder the bending deformation of the fish body, the progressive interlocking of the bony plates could increase the flexural stiffness of the fish body and provide functions similar to the tendon effect (energy storage/release), thus improving the swimming efficiency of the fish [10]. In the tests of the dermis-cut samples, it was observed that the *M*-*C* curves moved significantly to the right, which means that the flexural stiffness of the fish body was significantly reduced. The results proved that s. compactum in fish skin had a critical effect on flexural stiffness. Therefore, some factors probably affect the bending deformation properties of the sturgeon fish body, such as s. compactum, body shape, and bony plates.

In the process of the undulatory locomotion of fish, the s. compactum can act as a whole-body external tendon and significantly promote muscle contraction during swimming [12]. Although the bending process of the fish body only involves the elastic deformation of the fish skin, the analysis of the fracture mechanism of fish skin may help us further understand the flexural response of the fish. Fish skin with a very low mineralization degree showed excellent ductility and flexibility. For bent fish, the skin on the convex side was stretched. Because the tensile loads required by the fish skin in the elastic deformation stage were minimal, the resistance in the bending process of the fish body was also minimal, and the energy consumed was deficient. Furthermore, the skin on the inner concave side of the bent fish was compressed, and mechanical energy could be stored in the form of strain energy in the s. compactum, which could effectively promote the subsequent swimming motion.

## 5. Conclusions

This paper comprehensively studied the surface morphology, fracture performance, and bending properties of Chinese sturgeon fish skin. The tests performed in this study further extended our understanding of sturgeon fish skin’s toughening mechanism and potential tendon effect. The following specific conclusions can be drawn:

(1) Some tiny placoid scales are distributed on the skin surface of Chinese sturgeon. These scales are irregularly arranged and show considerably different structural morphology, indicating that they have degenerated tissues.
(2) The results of tensile fracture tests revealed that the skin of the Chinese sturgeon is a biological material with significant toughness characteristics, and its average fracture toughness is 22.67 ± 1.23 kJ/m^2^, which is even tougher than that of the general cortical bone. The superior mechanical properties of sturgeon fish skin can effectively help the Chinese sturgeon resist predator attacks and avoid damage to the fish body caused by large bending movements.
(3) The bending tests showed that flexural stiffness decreased gradually from the anterior region to the posterior region of the sturgeon fish body, which indicated that the posterior region of the fish body had higher flexibility. In addition, compared with the intact samples, the flexural stiffness of the samples (with scales removed) decreased slightly, and the decline in the posterior region of the fish body was more significant than in the anterior and middle regions. Compared with the intact samples and the samples with scales removed, the flexural stiffness of the dermis-cut samples was significantly reduced, indicating that the fish skin played a vital role in the flexural response of the fish body.
(4) Unlike the scales of most fish species, for Chinese sturgeon, there was no overlap between the lateral and ventral bony plates. The bending tests showed that when the posterior region of the fish body was significantly bent, the lateral bony plates would hinder the bending deformation of the fish body to a certain extent. The bony plates showed higher mineralization and deformation resistance compared to most fish scales. This means that the non-overlapping bony plates could effectively decrease the difficulty of fish deformation, thus reducing the energy consumed during bending deformation. This work provides a novel bionic template for exploring and designing flexible, protective, and locomotory systems.

At present, we have only conducted preliminary research on the biological protective properties of Chinese sturgeon skin, and there are still significant challenges in applying the biological functions of fish skin materials to engineering fields, such as limited applicability, scaling issues, material durability, and manufacturing complexity.

## Figures and Tables

**Table 1:** Average maximum tangent flexural stiffness at three locations of the fish body.

Sample	Locations/mm	Flexural Stiffness *EI*/N·m^2^		
		Intact	Scales Removed	Dermis Cut
Sample 1	60	0.047	0.044	0.028
	130	0.036	0.035	0.019
	250	0.021	0.018	0.007
Sample 2	80	0.045	0.043	0.026
	180	0.033	0.031	0.016
	290	0.016	0.013	0.003
Sample 3	100	0.044	0.042	0.023
	220	0.031	0.030	0.014
	340	0.017	0.014	0.004

## Data Availability

Data are only available upon request due to restrictions regarding, e.g., privacy and ethics. The data presented in this study are available from the corresponding author upon request. The data are not publicly available due to their relation to another ongoing research project.
